# Integrated Meta-Omics Analysis Unveils the Pathways Modulating Tumorigenesis and Proliferation in High-Grade Meningioma

**DOI:** 10.3390/cells12202483

**Published:** 2023-10-18

**Authors:** Deeptarup Biswas, Ankit Halder, Abhilash Barpanda, Susmita Ghosh, Aparna Chauhan, Lipika Bhat, Sridhar Epari, Prakash Shetty, Aliasgar Moiyadi, Graham Roy Ball, Sanjeeva Srivastava

**Affiliations:** 1 Department of Biosciences and Bioengineering, Indian Institute of Technology Bombay, Powai, Mumbai 400076, India; deeptarupbiswas2020@gmail.com (D.B.); halderankit530@gmail.com (A.H.); abarpanda1994@gmail.com (A.B.); aparna.chauhan278@gmail.com (A.C.); 2 Leibniz-Institut für Analytische Wissenschaften—ISAS, 44227 Dortmund, Germany; susmitaghosh191996@gmail.com; 3 Department of Biological Sciences, Sunandan Divatia School of Science, NMIMS Deemed-to-be University, Mumbai 400056, India; lipika.u@gmail.com; 4 Department of Pathology, Tata Memorial Centre, Mumbai 400012, India; sridhep@gmail.com; 5 Department of Neurosurgery, Tata Memorial Centre, Mumbai 400012, India; drpmshetty@gmail.com (P.S.); aliasgar.moiyadi@gmail.com (A.M.); 6 Medical Technology Research Centre, Anglia Ruskin University, East Rd., Cambridge CB1 1PT, UK; graham.ball@aru.ac.uk

**Keywords:** meningioma, integrin-linked kinase (ILK), extracellular matrix organization (EMO), proteomics, transcriptomics, apoptosis, integrated-omics, meta-analysis

## Abstract

Meningioma, a primary brain tumor, is commonly encountered and accounts for 39% of overall CNS tumors. Despite significant progress in clinical research, conventional surgical and clinical interventions remain the primary treatment options for meningioma. Several proteomics and transcriptomics studies have identified potential markers and altered biological pathways; however, comprehensive exploration and data integration can help to achieve an in-depth understanding of the altered pathobiology. This study applied integrated meta-analysis strategies to proteomic and transcriptomic datasets comprising 48 tissue samples, identifying around 1832 common genes/proteins to explore the underlying mechanism in high-grade meningioma tumorigenesis. The in silico pathway analysis indicated the roles of extracellular matrix organization (EMO) and integrin binding cascades in regulating the apoptosis, angiogenesis, and proliferation responsible for the pathobiology. Subsequently, the expression of pathway components was validated in an independent cohort of 32 fresh frozen tissue samples using multiple reaction monitoring (MRM), confirming their expression in high-grade meningioma. Furthermore, proteome-level changes in EMO and integrin cell surface interactions were investigated in a high-grade meningioma (IOMM-Lee) cell line by inhibiting integrin-linked kinase (ILK). Inhibition of ILK by administrating Cpd22 demonstrated an anti-proliferative effect, inducing apoptosis and downregulating proteins associated with proliferation and metastasis, which provides mechanistic insight into the disease pathophysiology.

## 1. Introduction

Meningioma, the most common primary intracranial tumor, is predominantly slow-growing and known to arise primarily from arachnoid cap cells [1]. Meningioma has broadly been classified into 3 grades, majorly relying on histopathology and cytomorphological criteria. Grade I (benign) tumors comprise 95% of total meningioma cases, grade II (atypical) tumors comprise 4–5%, and, grade III (anaplastic) tumors comprising less than 1% are fast-growing malignant tumors [2]. Benign WHO grade I meningioma, which accounts for 80% of all meningioma cases, may be surgically excised. In contrast, high-grade meningioma, which accounts for the remaining 20% of cases, provides significant treatment hurdles and is becoming the core of research interest due to its high recurrence rate and aggressiveness [3,4,5]. Depending on the tumor’s location and degree of brain invasion, surgical excision might be challenging, while the substantially enhanced risk of recurrence has led to the recommendation of radiotherapy as an adjuvant therapy after resection [6,7,8]. Chemotherapy is resorted to when no other surgical or radiation treatments are available, but it is still experimental and under clinical trials. The effectiveness of chemotherapy and radiation is limited and prone to toxic side effects, making the condition challenging to treat [9,10]. All of these aspects warrant investigating the development of better strategies to contain the explosive growth of high-grade meningioma and achieve better prognostic outcomes.

Global initiatives and advancements in clinical exploration have developed the International Consortium on Meningioma (ICOM) to promote and drive meningioma research [11]. In the last 10 years, different studies on meningioma have revealed new insights and improved the understanding of the progression of the disease [12,13]. Genomics studies reported that *NF2* variants are strongly associated with meningioma, wherein aberrant expression of Merlin protein leads to its transfer from the plasma membrane to the nucleus and results in alteration of cell adhesion and contact inhibition [1,14]. Although NF2 involvement is one of the important findings in meningioma, the frequency and occurrence are restricted to around 40% of total tumors [15,16]. Other non-*NF2* mutational signatures that are prevalent in meningioma include TRAF7, AKT1, POLR2A, PIK3CA, KLF4, SMARCE1, and BAP1. Causative variants in SMO, AKT1, and the TERT promoter seem to indicate increased risk of tumor recurrence and might be indicators for close surveillance [17]. Recently, another study reported mutations in ARID1A and SMARC genes that constitute the SW1/SNF chromatin remodeling complex [18]. In addition to different genomics studies, there have also been transcriptome and proteome-wide studies to identify biomarkers and altered biological pathways in meningioma. Mass spectrometry-based proteomics has widened the possibility of pinpointing biomarkers of various subtypes through high-throughput data generation and validation. In the last 5 years, several proteomics studies have reported proteome-level alterations and associated biological pathways in meningioma [19,20,21]. Among these studies, Mukherjee et al. (2020) and Dunn et al. (2019) investigated perturbations between meningioma tumors and non-tumor control samples via grade-wise comparisons in fresh frozen tissue samples. Additionally, Papaioannou et al. (2019) investigated grade-wise comparisons in formalin-fixed paraffin-embedded (FFPE) tissues. These proteome-level investigations have enhanced the understanding of meningioma by identifying protein markers and also established the foundation for better therapeutic strategies.

Surgical excision is indeed the preferred and often effective therapy for meningioma; however, tumor location and recurrence make complete tumor resection a challenge [22,23]. Many studies have investigated the potential of molecular inhibitors in established meningioma cell lines like BEN-MEN-1 for lower grade meningioma and IOMM-Lee and CH157-MN for higher grade meningioma. A few of the inhibitors that have been tested and taken forward for clinical trials include the cyclooxygenase-2 inhibitor celecoxib, the histone deacetylase inhibitor AR-42, and the mTOR inhibitors sirolimus and everolimus [24]. Despite the acceleration in drug repurposing and therapeutic target identification, high-grade meningioma remains indecipherable.

In this study, we performed a meta-analysis of proteomics studies conducted by Mukherjee et al. (2020) and Dunn et al. (2019), and further integrated the results with transcriptomic datasets to understand the molecular level alterations in high-grade meningioma. The in silico pathway analysis was followed by validation of integrin-linked kinase (ILK) along with its associated pathway components, like ITGB1 and VIM, which are known to play important roles in regulating the EMO and EMT transmission responsible for the progression of meningioma. Furthermore, the role of ILK and its potential in tumorigenesis was investigated in the IOMM-Lee cell line, unveiling the regulatory effect of ILK on apoptosis, ECM, and tumor progression. The integrated proteomics-transcriptomics analysis, pathway enrichment mapping, and in vitro studies provide a better understanding of disease progression and the underlying mechanism of the pathobiology in high-grade meningioma.

## 2. Materials and Methods

### 2.1. Omic Data Mining, Literature Search, and Data Analysis

The data mining for the meta-analysis of meningioma was performed in three public data repositories: ArrayExpress, ProteomeXchange Consortium, Gene Expression Omnibus (GEO), and Omics Discovery Index (OmicsDI) for Proteomics and Transcriptomics [24,25,26,27]. The primary keyword was “meningioma” and the other search keywords were “tissue” along with “human” as a species filter. Subsequently, two proteomic datasets, PXD014852 and PXD007073, were matched with the meta-analysis criteria and downloaded. The transcriptomic dataset was searched primarily in OmicsDI and ArrayExpress. The keywords included “meningioma” AND omics type “transcriptomics.” Microarray data belonging to HG-U133A platform were selected. GSE43290 was found to match the criteria and files were accessed using the GEO database. In addition to this, the outputs of the published manuscripts by Dunn et al. (2020) and Papaioannou et al. (2019) were also included to understand the commonalities between the data [20,28].

The raw files of PXD007073 and PXD014852 were re-analyzed in MaxQuant (v2.0.1.0) to minimize cross-search engine variability. Raw files were processed within Label-Free-Quantification (LFQ) parameters, setting label-type as “standard” with a multiplicity of 1. The match between runs was selected. Trypsin was used for digestion with a maximum missed cleavage of 2. Carbamidomethylation of cysteine (+57.021464 Da) was set as the fixed modification, whereas oxidation of methionine (+15.994915 Da) was set as the variable modification. The false discovery rate (FDR) was set to 1% for proteins, PSM, and site decoy fraction to ensure high protein identification/quantification reliability. The decoy mode was set to “reverse,” and the minimum peptide length was kept at 7AA.

### 2.2. Pre-Processing, Normalization, and Statistical Data Analysis of Proteomic Datasets

Correlation analysis and unsupervised clustering of PXD014852 and PXD007073 were performed to understand the data quality. Dataset integration or cross-dataset normalization was not performed to prevent technical bias. The two datasets were median-normalized and separately imputed using the kNN algorithm. Significant differentially expressed proteins were identified using Welch’s *t*-test with a *p*-value ≤ 0.05. The common list of differentially expressed proteins was taken forward to draw a correlation plot between the two datasets. The analysis was visualized using correlation plots, scatter plots, heatmaps, volcano plots, and violin plots drawn using the visualization libraries in Python (https://www.python.org/, accessed on 15 April 2023), Metaboanalyst, Orange [29], and Morpheus [30,31].

### 2.3. Data Quality Check and Statistical Analysis of Transcriptomic Datasets

The CEL format files containing the microarray experimental data were downloaded in the R environment to perform the statistical analysis. The Bioconductor package arrayQualityMetrics was used to assess the technical quality of arrays, which was determined based on background values and various scaling factors [32]. The difference of high-grade meningioma against control samples, i.e., arachnoid tissues in GSE43290, was used to identify differentially expressed genes (DEGs). Identification of DEGs was performed using LIMMA, an R-based open-source software integrated within afflmGUI [33]. The cut-off for adjusted *p*-value was set at 5% to identify significant differentially expressed genes.

### 2.4. Protein–Protein Interaction Network (PPIN) and Pathway Enrichment Analysis

The list of common significant differentially expressed proteins (DEPs) between the datasets was taken forward as input in Metascape and g:GOSt of g:Profiler for pathway enrichment analysis [34,35]. Gene set enrichment analysis (borad.mit.edu/gsea, accessed on 20 April 2023) was performed for both proteomic datasets using the KEGG pathway database under canonical pathways (CP) from the Molecular Signatures Database (MSigDB). The number of permutations was set to 1000, and the metric for ranking genes was the signal-to-noise ratio [36]. Network enrichment analysis and disease gene enrichment analysis were performed in STRING and Networkanalyst, taking DisGeNET and DISNOR as the reference disease knowledge base [37,38,39,40].

### 2.5. Sample Preparation for Targeted Proteomics Analysis

The samples used in this study were approved by the Institutional Ethics Committee of the Advanced Centre for Treatment Research and Education in Cancer (ACTREC), Tata Memorial Hospital (TMH), Mumbai, India, and IIT Bombay (ACTREC-TMC IEC No. 149). The healthy samples as the control references were collected from NIHMANS Brain Bank. Around 50 mg of tissue was resected to extract the proteins from the tissue specimens using a lysis buffer consisting of 8 M urea, Tris HCl buffer, and a protease inhibitor cocktail (PIC) (Sigma Aldrich^®^, St. Louis, MO, USA, Catalogue Number: 539131). First, 50 µg of proteins was reduced with TCEP, and then the proteins were alkylated with iodoacetamide (IAA). The resulting reduced and alkylated proteins underwent enzymatic digestion using trypsin (Pierce, Thermo Fisher Scientific, Vilnius, Lithuania). Following an overnight incubation of 16 h at 37 °C, the digests were concentrated through vacuum drying and reconstituted in 0.1% (*v*/*v*) formic acid (FA). Desalting of the peptides was performed using in-house C18 stage tips, as mentioned in our previous study [41]. The desalted peptides were subsequently dried and reconstituted in 0.1% (*v*/*v*) FA.

### 2.6. Transition List Preparation and Data Acquisition for Targeted Proteomics Analysis

The transition list for the selected proteins was prepared using Skyline. The settings and protocol were followed as discussed in our previous manuscript with slight modifications [42,43]. Data acquisition was conducted using a TSQ Altis Mass Spectrometer (Thermo Fisher Scientific) coupled with an HPLC system (Dionex Ultimate 3000, Thermo Fisher Scientific). A 1 ug sample of peptide was injected and separated using a Hypersil Gold C18 column (1.9 μm, 100 × 2.1 mm, Thermo Fisher Scientific). MRM runs were performed with a flow rate of 450 µL/min, cycle time of 2 s, and resolution of 0.7 m/z (Q1 and Q3) across a 10 min LC gradient. The solvent system consisted of 0.1% formic acid (FA) and 100% acetonitrile (ACN). The obtained data were analyzed using Skyline-daily, as discussed in a previous study [44]. Peak selection and refinement were conducted by considering peak shape, dot product, and retention time.

### 2.7. Cell Culture, Stocks, and Doses of Inhibitor

The IOMM-Lee high-grade meningioma cell line was obtained from ATCC (CRL-3370). Cells were grown in DMEM supplemented with 10% fetal bovine serum, 100 units/mL penicillin, and 100 μg/mL streptomycin. Cpd22^®^ (Merck Millipore, Burlington, MA, USA) was dissolved in DMSO to prepare the drug solution. Cells were cultured in a CO_2_ incubator under standard conditions of 5% CO_2_, 95% humidity, and 37 °C temperature. Once the cells reached the desired confluency (70–80%), the cells were cleared by trypsinization and transferred to new culture vessels for maintenance.

### 2.8. Cell Proliferation Assay

Around 8000 cells were seeded in a 96-well plate and incubated until morphology was achieved. The drug was dissolved in DMSO, and various concentrations of the drug were added to the cells. Following incubation, 10 µL of 3-[4,5-dimethylthiazol-2-yl]-2,5 diphenyl tetrazolium bromide (MTT) reagent from the stock solution of 5 mg/mL was added, and the cells were incubated for another 3 h followed by the addition of 150 µL of DMSO to each well. The absorbance was measured at 570 nm.

### 2.9. Apoptosis Assay

Cells were grown to ~70% confluency, followed by Cpd22 treatment. The FITC Annexin V Apoptosis Detection Kit (BD Biosciences, Franklin Lakes, NJ, USA) was used to detect apoptosis, as discussed in our previous manuscript [45]. Cells were suspended in 1× binding buffer, followed by double staining with FITC Annexin V and PI. Each cell was analyzed through flow cytometry. The experiments were performed with biological replicates to determine the reproducibility.

### 2.10. Protein Extraction and Mass Spectrometry Data Acquisition

The cell pellets of three biological replicates from Cpd22 treatment and control samples (DMSO-treated) were gently washed with 1× phosphate-buffered saline (PBS) and lysed using urea (8 M) buffer. Around 300 μL lysis buffer containing 8 M urea, 50 mM Tris pH 8.0, 75 mM NaCl, 1 mM MgCl_2_, and 500 units of benzonase was added to the cell pellets followed by a brief sonication on ice to produce the cell extract. The debris was separated by centrifugation at 8000 rpm for 15 min at 4 °C. The supernatant was collected in a fresh sterile Axygen/microcentrifuge tube. Protein quality was checked by performing SDS-PAGE, and simultaneously, the concentration of protein in the tissue lysate was determined using Bradford Assay. Approximately 100 ug of protein sample was taken for tryptic digestion. Prior to digestion, the proteins were reduced with 20 mM of TCEP, followed by alkylation with 37.5 mM of IAA. The digested peptide was then vacuum-dried and reconstituted in 0.1% (*v*/*v*) FA. To reduce the salt concentration in the digested peptide, it was cleaned up using C-18 stage tips by obeying the reverse-phase column chromatography principle. The cleaned peptide was further dried and dissolved in 0.1% (*v*/*v*) formic acid (FA). The peptide concentration was calculated using the Scopes method from its O.D. values at 205 nm and 280 nm (33). A 1 µg sample of the peptide was analyzed using the Orbitrap Fusion Tribrid Mass Spectrometer (Thermo Fischer Scientific) equipped with the Easy-nLC 1200 system, with a gradient of 80% ACN and 0.1% FA for 120 min and a flow rate of 300 nL/min. Mass spectrometric data acquisition was performed in data-dependent acquisition mode with a mass scan range of 375–1700 m/z and mass resolution of 60,000. For dynamic exclusion under MS (precursor ions), the mass tolerance was set to 10 ppm with an exclusion duration of 40 s. The ddMS^2^ (data-dependent MS^2^) scan properties activation type was set to HCD with the collision energy mode fixed to 30%, and spectra were acquired on an Orbitrap with a resolution of 15 k and maximum injection time of 30 ms.

### 2.11. Label-Free Quantification and Biological Analysis of Cell Line Proteome Data

The proteomic raw files were analyzed using MaxQuant with similar parameters as were used for the meta-analysis [46]. Furthermore, proteins without any missing values were taken for differential proteomics analysis of the control and treatment groups. The normalized and log2-transformed data were used to calculate fold change, *p*-value, and FDR. The *p*-value were set to ≤0.05 for the significance level. The list of significant differentially expressed proteins (DEPs) and proteins found in either of the two groups (binary proteins) was further analyzed in Reactome and Metascape to understand the enriched biological pathways [34,47]. The protein–protein interaction network was constructed in STRING [37]. Finally, the altered pathways and their components in the meta-analysis were compared with cell line data to assess the influence of ILK inhibition on key components of the signaling cascades that were also overexpressed in meningioma.

### 2.12. In Silico Drug Docking of Compounds Similar to Cpd22 as Well as FDA-Approved Drugs

The 3D crystal structure of ILK (PDB ID: 3 KMW) was downloaded from PDB (https://www.rcsb.org/, accessed on 27 May 2022) [48]. Protein remodeling was performed using Chimera [49]. It involved the following steps: removal of alpha-parvin (chain B), Mg, ATP, and water molecules, followed by energy minimization using the steepest descent algorithm for 50,000 steps. After this, the preprocessed structure was uploaded and visualized using NGL viewer [50]. The PDB structure of the protein was preprocessed before docking using an in-built tool present in the Galaxy webserver (https://cheminformatics.usegalaxy.eu/, accessed on 27 May 2022) [51]. The grid box parameters were calculated using Mg-ATP as a ligand with the help of the RDkit tool embedded within the Galaxy webserver (https://www.rdkit.org/, accessed on 27 May 2022). Molecular docking was performed on the Galaxy webserver using the AutoDock Vina tool with exhaustiveness set to 24 following calculation of the grid parameters using RDkit. The parameters were 14.757, 21.972, and 18.3 for the x-, y-, and z-axes, respectively. The center_x, center_y, and center_z values were −7.9295, 3.039, and 11.623, respectively. The receptor–ligand interactions were checked on Discovery Studio Visualizer.

The drug library was prepared by searching the CHEMBL database in the Galaxy web server, setting the Tanimoto cut-off score for similarity to 60. Further, the drug-likeness of compounds was studied using the QED tool [52]. The FDA-approved drugs were chosen on the basis of being antineoplastic and immunomodulating agents (https://www.whocc.no/atc_ddd_index/, accessed on 10 May 2022). The specific compounds were searched using specific parameters on CHEMBL (https://www.ebi.ac.uk/chembl/, accessed on 12 May 2022), like the maximum clinical phase trial or clinical phase trial 4, and to be reported as protein-kinase inhibitors. Also, a compound belonging to antipsychotic classification (https://www.whocc.no/atc_ddd_index/, accessed on 10 May 2022) having target molecule as serine/threonine kinase inhibitors reported in CHEMBL was chosen for docking with ILK. The compounds were pre-processed using the receptor-ligand tool present in the Galaxy web server before being subjected to docking.

## 3. Results

### 3.1. Integrated Omics Analysis of Meningioma Datasets

**Figure 1:**
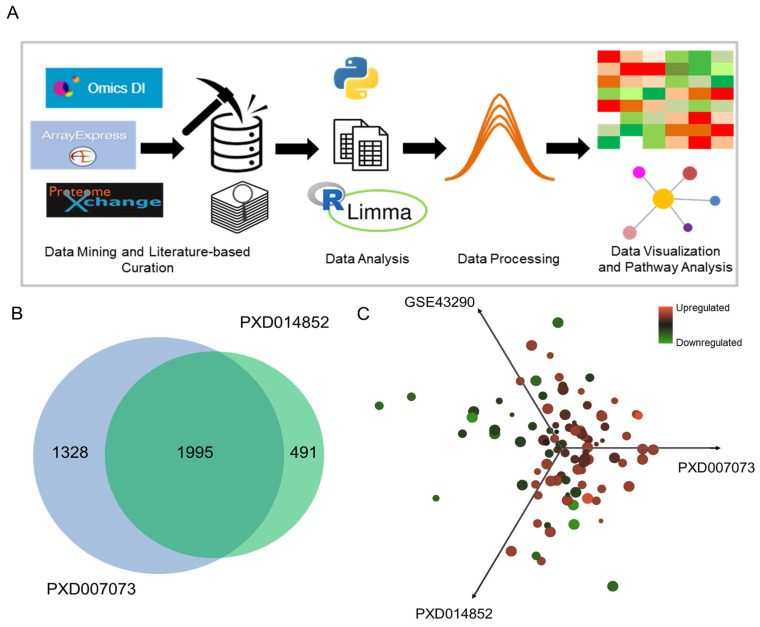
Integrated omics analysis to understand the commonalities driving disease progression in high-grade meningioma: (**A**) illustrates the schematic workflow of the proteomics and transcriptomics analysis of high-grade meningioma; (**B**) depicts the Venn diagram of common proteins between the two proteomic datasets, PXD007073 and PXD014852; (**C**) deciphers the correlation of common differentially expressed features based on scaled log2FC acquired from the statistical analysis.

The proteomic and transcriptomic datasets mined from the data repositories were re-analyzed, and the schematic outline of the meta-analysis is shown in Figure 1A and Table S1. A total of 1995 proteins were found to be common between the two datasets, which are shown in the form of a Venn Diagram (Figure 1B). Classification and fold change between the control and high-grade meningioma sample cohorts were performed to understand similarities in the trends of proteins between the datasets (Figure S1A). The commonalities between the two datasets, PXD014852 and PXD007073, led to the identification of around 248 differentially expressed proteins (Figure S1B). Transcriptomic data integration with proteomic data resulted in a total of around 1832 common features and 95 common concordant features with similar trends, which were taken forward to perform correlation analysis between the log2 fold change (Figure 1C and Figure S1C). A list of 38 significant (*p*-value < 0.05) common DEPs with similar trends was found between the proteomic and transcriptomic datasets, wherein myosin heavy chain 11 (MYH11), calponin 1 (CNN1), and glycoprotein M6A (GPM6A) were downregulated with minimal fold change; however, heterogeneous nuclear ribonucleoprotein U-like protein 1 (HNRNPUL1), filamin B, beta (FLNB), and GDP-mannose 4,6-dehydratase (GMDS) were upregulated with maximum fold change (Figure S2A). This list of DEPs was taken forward for biological pathway mapping and enrichment analysis.

### 3.2. Pathway Mapping and Gene-Set Enrichment Analysis to Understand the Proteomic and Transcriptomic Alterations in High-Grade Meningioma

**Figure 2:**
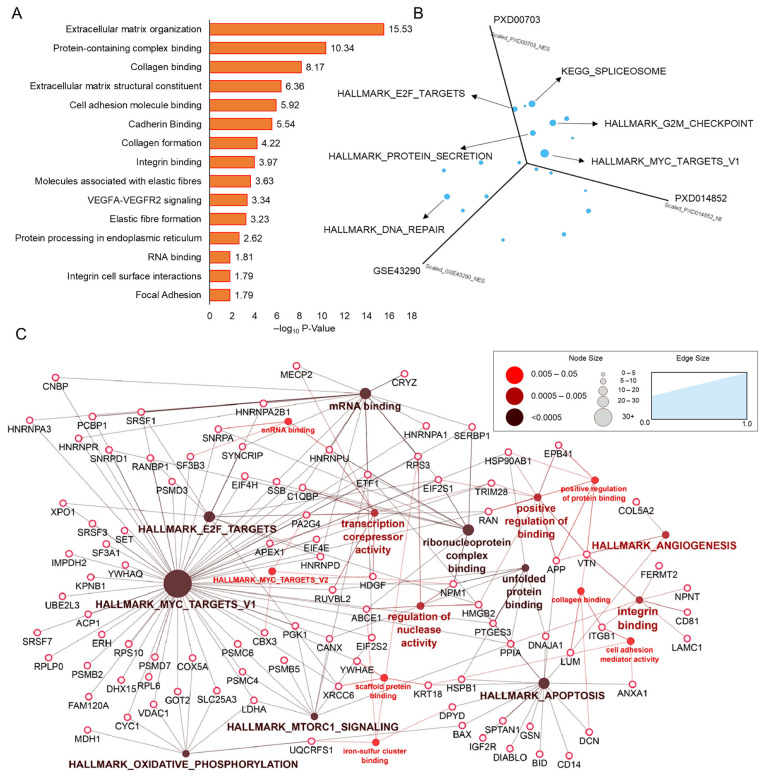
Biological interpretation of the common alterations in high-grade meningioma: (**A**) deciphers the enrichment of altered pathways using common dysregulated proteins; (**B**) shows the linear projection plot illustrating common gene sets between the three datasets with positive correlations with high-grade meningioma; (**C**) depicts the network analysis of genes and proteins involved in high-grade meningioma tumorigenesis and proliferation.

A list of common dysregulated proteins was taken forward to perform pathway mapping using over-representation analysis. Extracellular matrix organization (R-HSA-1474244) was found to map with the highest score, followed by collagen binding, cell adhesion, integrin cell surface interactions, and the VEGF signaling pathway, as shown in Figure 2A and Figure S2B,C. The results of gene set enrichment analysis showed a total of 17 common gene sets with positive correlations with high-grade meningioma, including MYC target variant 1 (M5926), a hallmark gene set of proliferation that has been found to be most prominent in cancers with a maximum consolidated normalized enrichment score (NES), followed by E2F targets and the cell cycle G2/M checkpoint, which also came under the proliferation category of the hallmark gene set (Figure 2B). In addition, the spliceosome, DNA repair, and protein secretion were also found to be common pathways mapped with all three datasets. All of these pathway components were integrated together to construct a network for better visualization and interpretation of the connections (Table S2 and Figure 2C).

### 3.3. Validation of the Potential Common Markers Using MRM-based Targeted Proteomics

A total of 32 fresh frozen tissue samples, including 13 high-grade (Grades 3 and 2), 10 low-grade (Grade 1), and 9 control (arachnoid and dura tissues) samples, were selected for the study (Table 1). A list of unique peptides, including vimentin (VIM), integrin subunit beta 1 (ITGB1), integrin-linked kinase (ILK), prelamin-A/C (LMNA), fibrinogen gamma chain (FGG), and heat shock protein HSP 90-alpha (HSP90AA1), were taken forward and optimized for the development of MRM-based targeted validation (Table S3).

**Figure 3:**
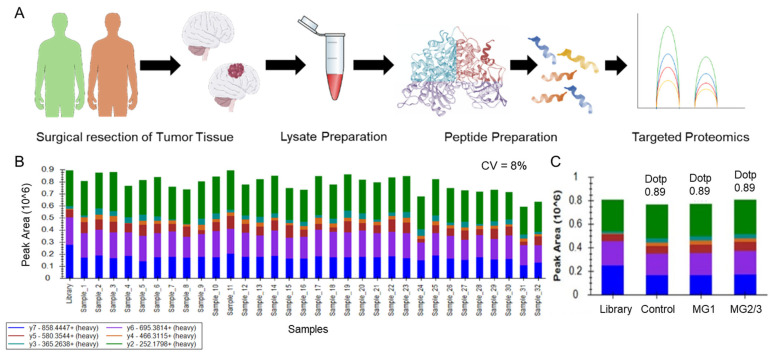
Experimental setup and quality assessment of targeted proteomics validation: (**A**) illustrates the schematic workflow of targeted proteomics-based biomarker validation in meningioma tissues; (**B**,**C**) depicts the comparison of peak intensities of a standard non-human spike-in heavy labeled peptide “VFPYDNTLPK” added to each samples to monitor the run-to-run variation sample-wise and group-wise, respectively. No significant variation in spike-in peptide intensity was observed, ruling out run-to-run variation. [MG = Meningioma].

A schematic outline of the experiment is illustrated in Figure 3A. The coefficient of variance (CV) of a spiked synthetic peptide to monitor the variation between runs was found to be 8%, and the peak areas of the three conditions are shown in Figure 3B,C.

**Figure 4:**
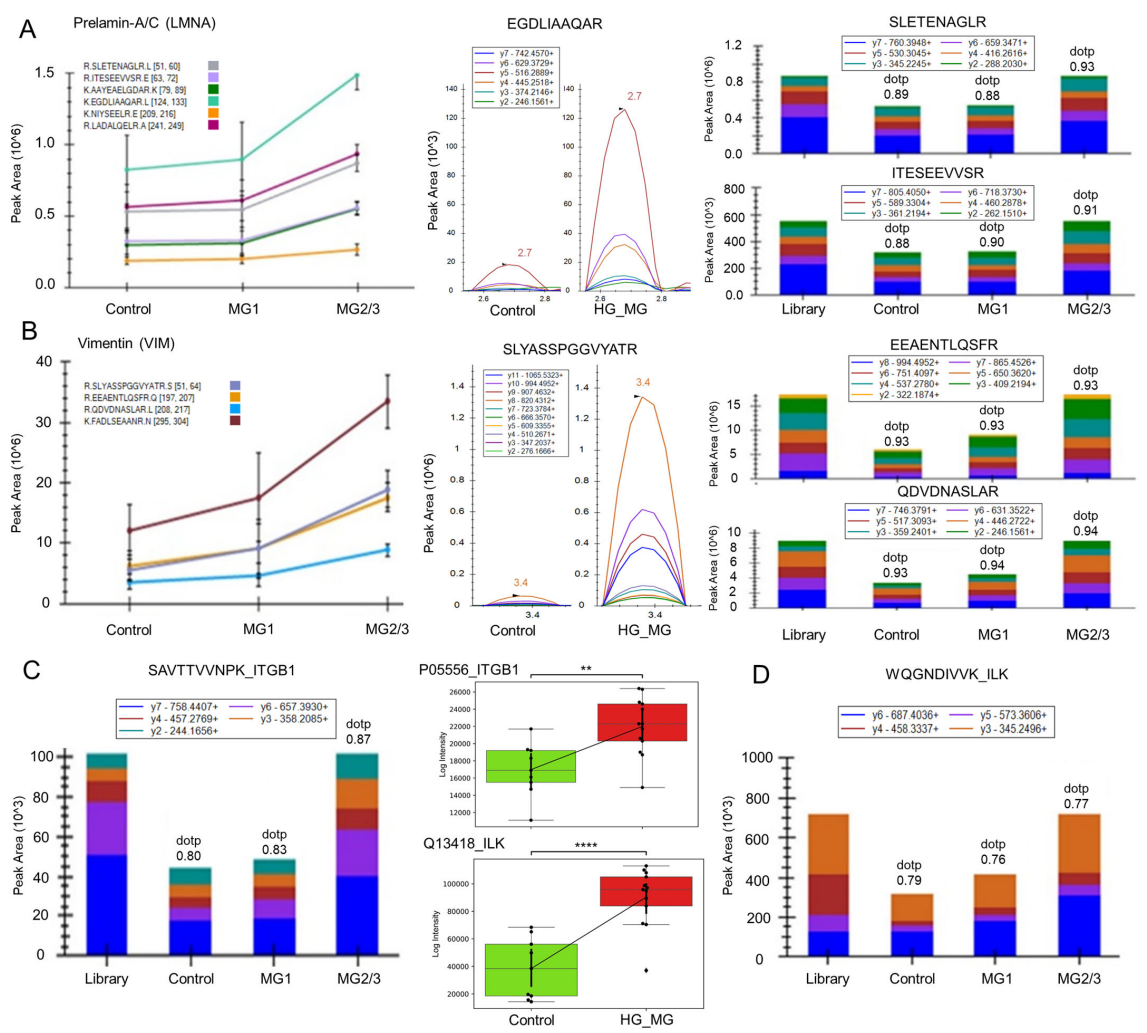
Validation of key proteins in altered pathways using MRM-based targeted proteomics depicting peptide-level intensities and peak areas: (**A**) prelamin-A/C (LMNA), (**B**) Vimentin (VIM), (**C**) integrin subunit beta 1 (ITGB1), and (**D**) integrin-linked kinase (ILK). The plots were made with peak areas, with the significance level calculated based on the independent *t*-test with Bonferroni correction (*p*-value annotation in legend: **, 1.00 × 10^–03^ < *p* ≤ 1.00 × 10^–02^; ****, *p* ≤ 1.00 × 10^–04^). [MG = Meningioma; HG = High Grade; LG = Low Grade].

LMNA and VIM were found to be upregulated in high-grade meningioma for the peptides monitored (Figure 4A,B). The peak areas of peptides ITGB1 and ILK showed upregulation in high-grade meningioma when compared with control samples and low-grade meningioma (Figure 4C,D).

### 3.4. In Vitro Inhibition Using Cpd22 Reveals the Anti-Tumor Potential of the Pathway

**Figure 5:**
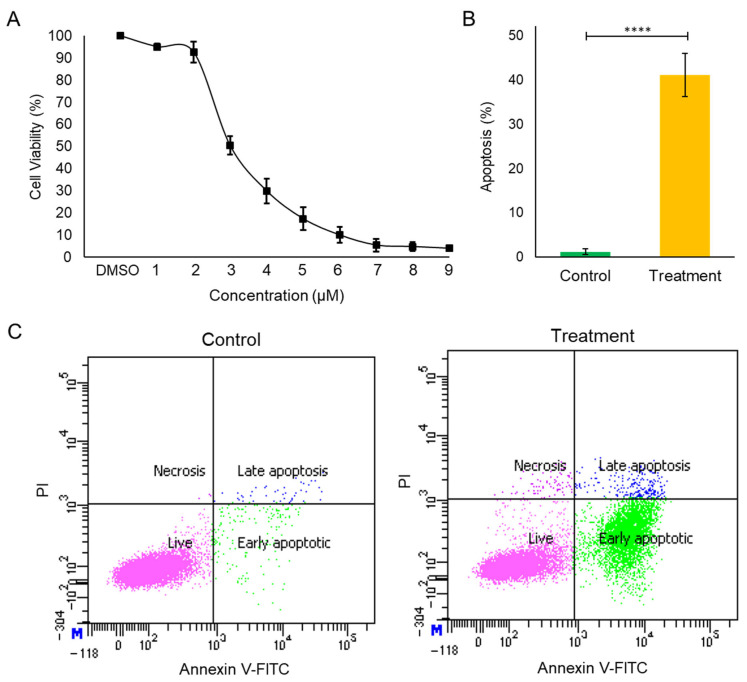
Impact of Cpd22 in proliferation and apoptosis. (**A**) Determination of IC-50 of Cpd22 in the IOMM-Lee cell line; morphology after 24 h treatment with DMSO and Cpd22; (**B**) Percentage of apoptotic cells after 24 h treatment with DMSO and Cpd22 determined by flow cytometry with t-statistic (****, *p* ≤ 1.00 × 10^–04^); (**C**) Flow cytometry assay: the fraction of apoptotic cells was determined by flow cytometry with Annexin V–FITC and Propidium Iodide (PI) double staining.

ILK, being the central signaling hub along with overexpression in high-grade meningioma, regulates pivotal cellular homeostatic processes, including cell growth, proliferation, cell survival, etc. The importance of ILK in homeostatic processes was examined through treatment with a potential ILK inhibitor in the high-grade meningioma cell line, IOMM-Lee. Cpd22 treatment for 24 h at various doses, including 0, 1, 2, 3, 4, 5, 6, 7, 8, and 9 µM, decreased cell growth in a concentration-dependent manner. The reduction in half of the cell population (IC_50_) was identified in the range of ~ 3 to 4 µM (Figure 5A). The 3 µM concentration of the drug was taken for further cell assays. The apoptotic potential of ILK inhibition was studied by staining the cells using the FITC-Annexin V assay. Following treatment, the cells were stained with FITC-Annexin V and PI and the amount of apoptosis was quantified through flow cytometry. A substantial change in apoptotic morphology was seen in the treated population. In the treated population, ~42% of cells were found to be in the apoptotic phase. The output of the flow cytometry-based apoptosis assay was interpreted with biological replicates to calculate the standard deviation (Figure 5B,C).

### 3.5. Proteomic Alterations and Biological Pathway Perturbations Post-Treatment with an ILK Inhibitor in the Meningioma Cell Line

**Figure 6:**
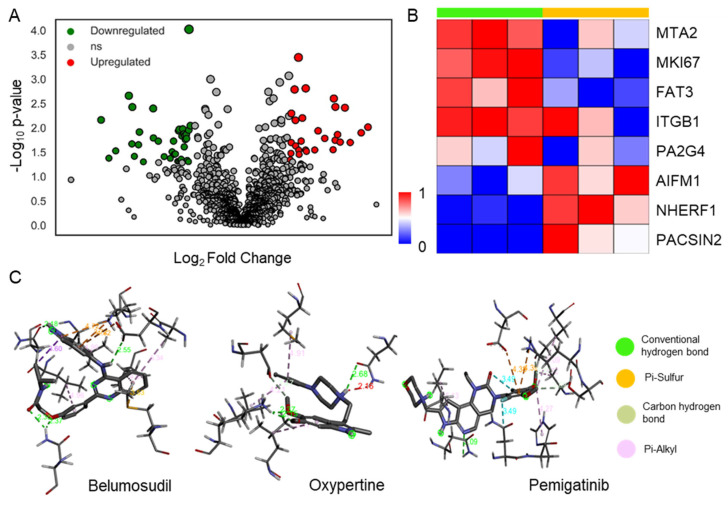
Role of Cpd22 in proteome-level regulation and drug discovery: (**A**) deciphers the differentially expressed proteins in Cpd22-treated cells in comparison to control cells, taking fold change as 2 and a *p*-value cutoff of 0.05 using proteomics analysis; (**B**) represents the heatmap showing expression changes in treatment and control groups on a scale of 0–1; (**C**) shows the drug docking results along with binding affinity (kcal/mol) scores of belumosudil, oxypertin, and pemigatinib.

MaxQuant analysis of six files, which included three biological replicates for control and Cpd22-treated cells, provided a total of 1721 proteins. The correlation between the samples was found to be greater than 0.8, and the intensities were properly normalized (Figure S3A,B). Principal component analysis (PCA) showed two clear clusters of the control and treatment groups, with 33.3% as PC1 and 21% as PC2 (Figure S3C). The data analysis resulted in 245 binary proteins (identified in either of the two conditions) and 66 significant differentially expressed proteins, as shown in Figure 6A. The enriched pathways with differentially expressed proteins in the treatment group compared to the control group showed alterations in focal adhesion (hsa04510), regulation of actin cytoskeleton (hsa04810), the PI3K-Akt signaling pathway (hsa04151), and the spliceosome (hsa03040). In addition to the pathway analysis, a few key proteins were found to be altered in the treatment group, like metastasis associated 1 family member 2 (MTA2) and marker of proliferation Ki-67 (MKI67), were found to be downregulated in the Cpd22-treated population. However, Na+/H+ exchanger regulatory factor 1 (NHERF1) and protein kinase C and casein kinase substrate in neurons (PACSIN2) were found to be upregulated in the Cpd22-treated population. A heatmap depicting a list of eight interesting proteins with functional correlations is shown in Figure 6B.

### 3.6. Screening of Compounds Using in Silico Docking for ILK

The results from in silico screening of FDA-approved drugs showed that some drugs have high binding affinity for ILK as well as other protein kinase inhibitors. Amongst these drugs, belumosudil, a known inhibitor of Rho-associated protein kinase 2 (ROCK2), was observed to have a high binding affinity of −9.904 kcal/mol. The receptor-ligand interaction showed several conventional hydrogen bonds with the following residues: ASN200, ASN279, and ASP339 (Figure 6C). In addition, compounds like selpercatinib, pemigatinib, and oxypertine showed high binding affinities of −8.570 kcal/mol, −8.111 kcal/mol, and −7.981 kcal/mol, respectively. Their interactions with residues of the target protein, ILK, were observed to form different types of bonds, such as conventional hydrogen bonds, pi–alkyl bonds, carbon-hydrogen bonds, alkyl bonds, pi–cation bonds, etc., which are shown in Figure 6C and Table S4. Some other notable small compounds exhibiting potential inhibition of ILK, along with drug-likeness analysis, are shown in Table S4. The reported drugs are to be further checked out in in vitro and in vivo based studies to understand their efficacy.

## 4. Discussion

Meningioma and its pathobiology have consistently garnered attention in neuroscience primarily due to its prevalence, although its aggressiveness and tendency to metastasize are comparatively lower than those of glioblastoma multiforme (GBM). Despite low-grade meningioma generally having a favorable prognosis, the recurrence and recent reports of metastasis in high-grade meningioma highlight its grave nature and severity [53,54]. With the upsurge in clinical research and advancements in omics technology and big data analysis, in-depth investigations have assisted in enhancing our understanding of meningioma pathobiology [28,55]. However, investigation and in-depth understanding of high-grade meningioma tumorigenesis and research with therapeutic potential are limited. Meningioma treatments solely depend on surgical resection and radiation therapy due to the lack of efficacy in chemotherapy. However, these clinical management strategies have limitations, particularly when dealing with advanced grades of meningioma or complications with tumor location [56,57]. These limitations and pitfalls could be resolved with a pellucid understanding of the molecular pathways involved, accelerating the discovery of targeted drugs for managing complicated clinical cases. An overview of early investigations and different clinical reports showed that altered expression of EGFR, PDGFR, and VEGFR in meningioma, which is one of the most important aspects of carcinogenesis, has been widely studied [58,59]. A plethora of studies and clinical trials have investigated the potential role of kinase inhibitors, including imatinib, vatalanib, and erlotinib; however, the paradox of disease progression, especially in high-grade meningioma, remains unclear.

**Figure 7:**
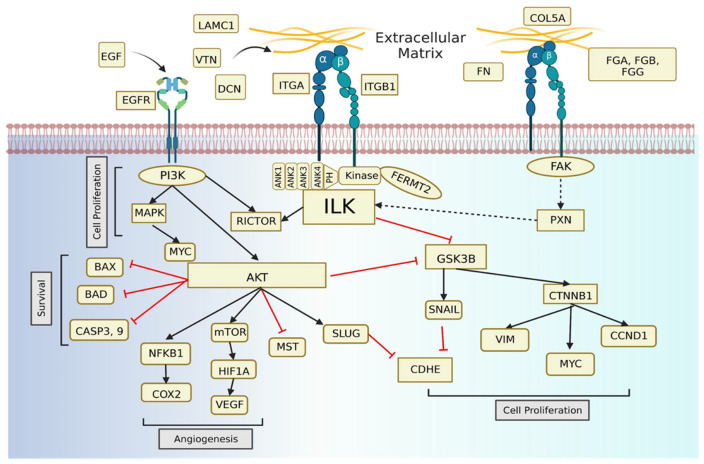
Summarization of the involvement of extracellular matrix organization (ECMO) and integrin in the regulation of altered biological pathways and signaling cascades in high-grade meningioma tumorigenesis. Cross-talk between components of these pathways with integrin-linked kinase (ILK) deciphers its potential as a master regulator. ILK results in the phosphorylation of GSK3B and PI3K, which triggers beta-catenin expression and activation of AKT, leading to cell proliferation, migration, and angiogenesis, respectively. Activation of the PI3K pathway also leads to increased expression of vascular endothelial growth factor (VEGF). Inhibition of ILK leads to arrest of phosphorylation, followed by decreased VEGF expression, inducing cell apoptosis. The positive feedback loop of PI3K/Akt/mTOR/c-Myc/mtp53 is also impacted, resulting in cells remaining arrested in the G1 phase of the cell cycle, leading to an anti-proliferative effect. [Created with BioRender.com].

The introduction of integrated omics analysis and meta-analysis has garnered tremendous improvements in comprehending the underlying mechanisms of dreadful brain tumors. This study used a meta-analysis pipeline to understand alterations in high-grade meningioma by integrating proteomic and transcriptomic datasets. Re-analysis and interpretation of publicly available proteomic datasets provided a total of 1995 proteins, which were further integrated with transcriptomic data to illustrate the perturbations between high-grade meningioma and reference controls. Our analysis resulted in concordant and discordant differential expression between datasets, which provided clues for comprehending the clinical complexity and biological correlations reported earlier [28]. GSEA and in silico pathway analyses of the integrated data unraveled the complexity by identifying alterations in signaling cascades in advanced grade meningioma, which primarily included extracellular matrix organization, integrin binding pathways, the spliceosome, and ECM proteoglycans on the one hand, and hallmark gene sets like apoptosis, angiogenesis, E2F, and Myc targets on the other. Functional clustering analysis and literature mapping aided in understanding the potential role of integrin-linked kinase (ILK) in regulating the altered biological cascades in high-grade meningioma, interconnecting integrin binding, angiogenesis, proliferation, and apoptosis (Figure 7). The inhibitory effect of ILK and control of tumorigenesis has been reported in regard to glioblastoma, but understanding its potential in high-grade meningioma remains unexplored [60].

The commonalities in the over-representation analysis indicated the association of integrin binding pathways, which regulate extracellular matrix organization (EMO), integrin cell surface interactions, and cell adhesion. These pathways were found to be upregulated in high-grade meningioma, with concordant proteome-level overexpression of integrin, heat shock proteins, fibrinogens, and lamins. The peptide-level validation of ITGB1 and lamin A/C in an independent sample cohort in this study, along with Annexin A1 validation in our previous study, confirmed that upregulation of these proteins, along with other cell-adhesion components, plays an important role in high-grade meningioma tumorigenesis inducing angiogenesis [21]. Conversely, advanced meningioma tumors exhibit stiffness, which also plays an important role during resection [61]. Nuclear lamins have a strong association with stiffness and have also been reported as a candidate biomarker for glioblastoma multiforme. Additionally, a previous study also found a connection between aggressiveness and the expression of lamins [62]. In our study, the expression of lamins was found to be upregulated in high-grade meningioma, reconfirming the connection with aggressiveness. Molecular function ontology enrichment analysis depicted the spliceosome and its associated biological pathway as one of the common alterations in both the proteomic and transcriptomic datasets. Bordeleau et al. reported the relationship between stiffness in ECM with mRNA splicing in cells [63]. The direct connection between ECM stiffness and SR splicing factors uses the integrin pathway, which in turn involves PI3K/AKT components to mediate biochemical signals [63]. High-grade meningioma shows higher levels of active AKT, and its phosphorylation inhibits proapoptotic factors like Bad, Bax, caspase-9, and forkhead, thereby inhibiting apoptosis [19]. The downregulation of Bax and Bid in the proteome-level meta-analysis formed a link between the upregulation of integrin components and tumorigenesis, with alterations in the apoptosis pathway. Additionally, the gene set enrichment analysis of the proteomic and transcriptomic datasets showed that Myc, E2F, and the G2M checkpoint hallmark gene sets were under the proliferation category and were found to have positive correlations with high-grade meningioma. Overall, the in silico pathway analysis and biological mapping unveiled overexpression of the integrin binding pathway, extracellular matrix organization (EMO), and angiogenesis in atypical and anaplastic meningioma, along with a positive correlation with proliferation gene set cascades. The upregulation of vimentin, a prominent marker of mesenchymal transmission in the validation experiment, indicated the likelihood of invasiveness with the advancement of grade [64]. This combination contributes to the poor prognosis associated with high-grade meningioma. Additionally, underexpression of pro-apoptotic proteins hinders cell death, further complicating treatment and cure.

Integrin-linked kinase (ILK), known to regulate a several downstream signaling cascades, connected all of the discussed altered pathways, acting as a master regulator. In this study, we validated the overexpression of ILK in high-grade meningioma using a MRM-based targeted proteomics approach. These results and correlation strengthen the involvement of integrin–cell adhesion in proliferation and angiogenesis. ILK also coordinates epithelial–mesenchymal transition (EMT), which acts as a contributing factor in the metastatic and invasive nature of anaplastic meningioma. Moreover, the inhibition of ILK would inhibit the activation of AKT, leading to reduced cell growth, and may promote apoptosis [10,64]. The administration of Cpd22 (ILK inhibitor) in a high-grade meningioma cell line (IOMM-Lee) provided evidence supporting the therapeutic potential of this kinase. Treatment with the compound showed the oncogenic role of the kinase as inhibiting the pathway triggered dose-dependent cell cytotoxicity in meningioma cells. Around 3–4 μM of the compound showed an anti-proliferative effect along with the appearance of a change in morphology after treatment for 24 h. Flow cytometry staining using FITC and PI indicated a clear spike in apoptotic populations after Cpd22 treatment. Furthermore, the cell assays demonstrated a strong correlation with the comprehensive proteomic characterization of the Cpd22 treatment that showed downregulation of the marker of proliferation Ki-67 (MKI67) and proliferation-associated 2G4 (PA2G4) in the treated population. Ki-67 has proven to be a reliable indicator of proliferation and is widely used to investigate tumor heterogeneity and prognosis. The downregulation of this marker in treated cells suggested the effectiveness of ILK inhibition in impeding tumor growth [65]. Apoptosis-inducing factor (AIFM1), a pro-apoptotic protein, was found to be upregulated in the treated population in the proteomics analysis, which correlated with the increase in apoptosis. Mitochondrial AIFM1 translocates to the nucleus, where it induces DNA fragmentation and chromatin condensation following the induction of apoptosis. The proteomics analysis also showed downregulation of metastasis associated 1 family member 2 (MTA2) and protocadherin fat 3 (FAT3), suggesting clear inhibition of the metastatic modulators. MTA2 serves as a pivotal nexus for coordinating cytoskeletal organization and transcription, establishing a crucial connection between nuclear cytoskeletal dynamics and promoting cancer metastasis [66]. MTA2 also has the potential to interact with eukaryotic initiation factor 4E (EIF4E), leading to the positive regulation of Twist expression, a recognized master regulator of epithelial–mesenchymal transition (EMT) [64]. Conversely, protein kinase C and casein kinase substrate in neurons (PACSIN2) was found to be upregulated in the treatment group. It has been reported to have a negative correlation with malignancy in glioma, whereas PACSIN2 was found to be downregulated in meningioma tumors when compared with controls in the meningioma datasets of BrainProt [67,68]. The Cpd22-based inhibition of ILK was shown to be efficient in controlling meningioma cells through the regulation of crucial proteins and pathways linked to tumorigenesis and metastasis, establishing the therapeutic potential of targeting ILK with regard to the regulation of proliferation, angiogenesis, apoptosis, and metastasis in high-grade meningioma. The highlighted role of ILK in disease progression thus resulted in considering the molecule as a major target for determining potential drug candidates using in silico docking.

The molecular docking performed in this study is indicative of a futuristic approach to the treatment of high-grade meningioma using FDA-approved drugs. FDA-approved drugs like oxypertine, pemigatinib, selpercatinib, and belumosudil showed encouraging results with high binding affinities and can be further explored for their inhibitory effects on ILK and treating high-grade meningioma. Selpercatinib is used as an FDA-approved drug for use against thyroid and lung cancers [69]. It is a known receptor for tyrosine kinase. Pemigatinib is approved for the treatment of cholangiocarcinoma and it targets FGFR, whereas oxypertine is currently used as a medication for psychotic disorders [70]. Belumosudil, the drug shown to have the highest affinity for ILK in drug docking, is an FDA-approved drug for use against graft-versus-host disease and is known to target the polymerization of G-actin fibrils and inhibit the Rho-ROCK-MRTF pathway [71]. The pathway coupling ILK to ECM matrix in high-grade meningioma unveils the potential that could be unleashed using belumosudil to treat high-grade meningioma. The results of the in silico docking can be further investigated in cell lines and animal models to prove its efficacy in treating high-grade meningioma in the future. Future investigations might lead to unraveling the huge potential of ILK as a therapeutic target by taking these repurposed drugs along with reported inhibitors like QLT-0267 and OSU-T315 in the context of high-grade meningioma. An earlier investigation with QLT-0267 in glioblastoma xenograft models showed decreased PI3K/Akt activity, VEGF secretion, and apoptosis induction, hence interfering with most cancer hallmark pathways [66]. Similarly, OSU-T315 has been used as a B cell-specific therapeutic ligand in breast and prostate cancer cells [65]. Meningioma has many unknown pathological mechanisms that can be discovered through in-depth molecular research; in that light, ILK inhibitors are indeed an integral research tool.

In conclusion, this study illustrates the power of integrated meta-analysis of proteomic and transcriptomic datasets in deciphering global alterations in high-grade meningioma, which was used herein to identify alterations in signaling cascades in high-grade meningioma. The enriched altered pathways highlight the role of ILK as a master regulator in high-grade meningioma tumorigenesis, regulating apoptosis, proliferation, and angiogenesis. The investigation of ILK inhibition using Cpd22 in a high-grade meningioma cell line (IOMM-Lee) showed a decrease in proliferation and angiogenesis followed by an increase of apoptosis. This study provides valuable mechanistic insight into the pathophysiology of high-grade meningioma and may contribute to the development of targeted therapies for this disease.

## Figures and Tables

**Table 1:** Demographic table representing the sample information.

Variables	High-Grade	Low-Grade	Control
Age			
N (N miss)	13 (0)	10 (0)	9 (0)
Mean ± SD	45.3 ± 13.2	48.2 ± 10.7	49.7 ± 29.7
Min–Max	23–67	31–74	0.5–88
Median (IQR)	46.5 (45–53)	47 (40.5–57.5)	45 (28–85)
Gender (%)			
Female	6 (46.2)	7 (70)	1 (12)
Male	7 (53.8)	3 (30)	8 (88)

## Data Availability

The mass spectrometry proteomic datasets have been deposited in the MassIVE repository with the dataset identifier MSV000092770. Targeted Proteomics Data Availability: Username: PASS04833. Full URL: ftp://PASS04833:PA3845xst@ftp.peptideatlas.org/, submitted on 26 May 2023.

## Supplementary Materials

The following supporting information can be downloaded at: https://www.mdpi.com/article/10.3390/cells12202483/s1.
