# Supplementary material for: Integrated Meta-Omics Analysis Unveils the Pathways Modulating Tumorigenesis and Proliferation in High-Grade Meningioma
Source: Cells. 2023 Oct 18;12(20):2483. doi: 10.3390/cells12202483 (PMC10604908; doi:10.3390/cells12202483)
Supplement: Supplementary file 1 [file cells-12-02483-s001.zip › Supplementary Figures.pdf]

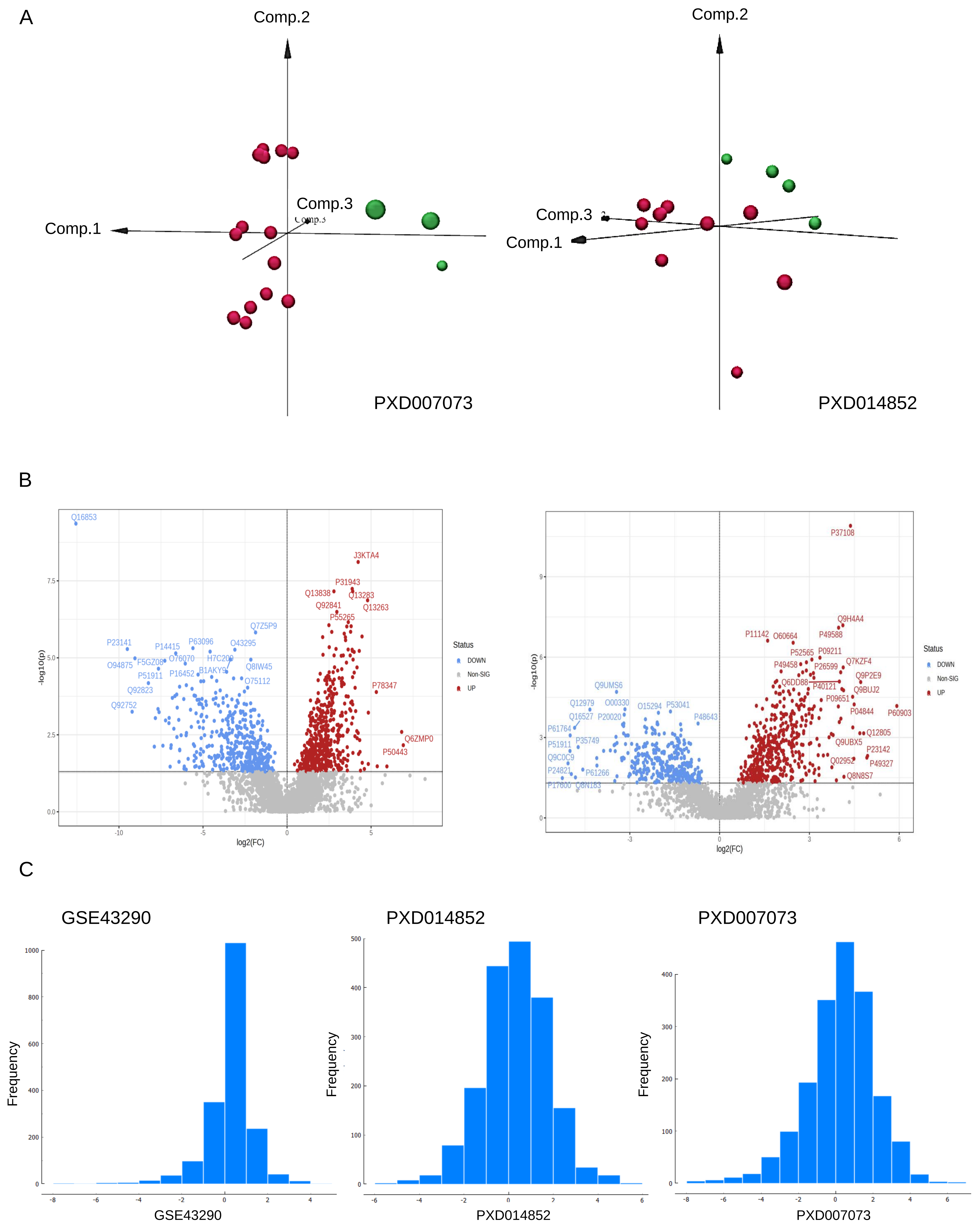
A
Comp.2
Comp.1
Comp.3
PXD007073
Comp.2
Comp.3
Comp.1
PXD014852
B
Status
DOWN
Non-SIG
UP
-log10(p)
log2(FC)
Q16853
J3KTA4
P31943
Q13838
Q13283
Q92841
Q13263
P55265
Q7Z5P9
P23141
P14415
P63096
O43295
O94875
F5GZ08
O76070
H7C200
Q8IW45
P51911
P16452
B1AKY9
O75112
Q92823
P78347
Q92752
Q6ZMP0
P50443
P37108
Q9H4A4
P11142
O60664
P49588
P52565
P09211
P49458
P26599
Q7KZF4
Q9P2E9
Q6DD88
P40121
Q9BUJ2
Q9UMS6
P09651
P04844
P60903
Q12979
O00330
O15294
P53041
Q16527
P20020
P48643
P61764
Q12805
P51911
P35749
Q9UBX5
P23142
Q9C0C9
Q02952
P49327
P24821
P61266
Q8N8S7
P17600
Q8N183
C
GSE43290
PXD014852
PXD007073
Frequency

Figure S1

A

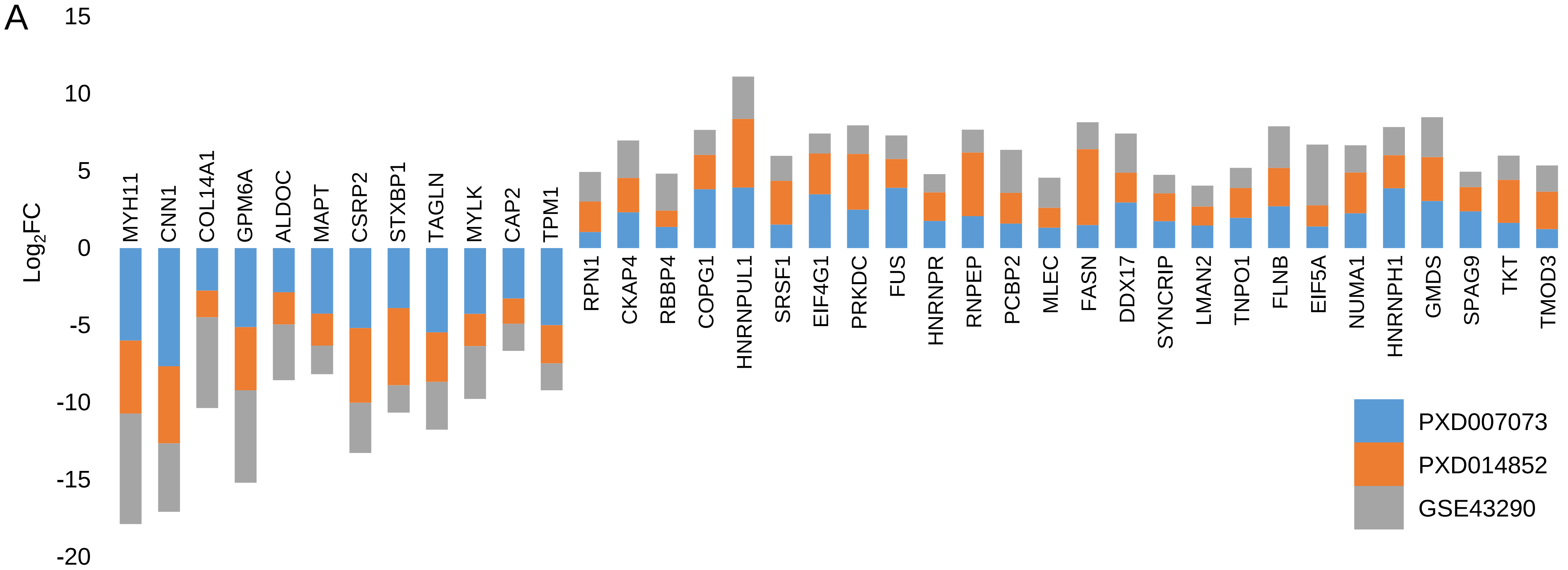

B

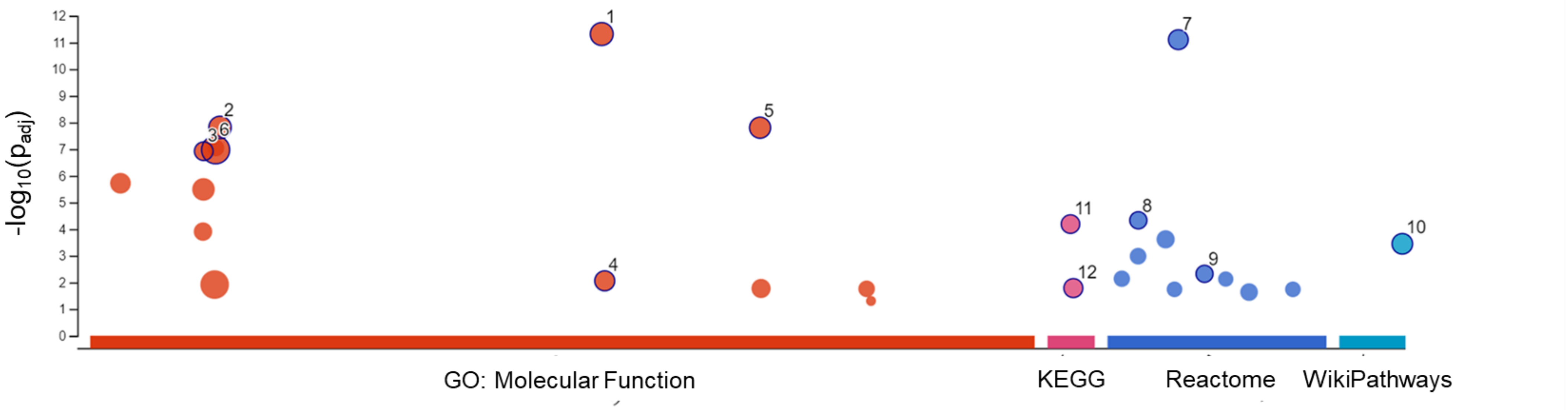

ID	Source	Term ID	Term Name	p_{adj} (query...
1	GO:MF	GO:0044877	protein-containing complex binding	4.797×10^{-12}
2	GO:MF	GO:0008092	cytoskeletal protein binding	1.529×10^{-8}
3	GO:MF	GO:0005201	extracellular matrix structural constituent	1.195×10^{-7}
4	GO:MF	GO:0045296	cadherin binding	8.747×10^{-3}
5	GO:MF	GO:0050839	cell adhesion molecule binding	1.569×10^{-8}
6	GO:MF	GO:0005515	protein binding	1.058×10^{-7}
7	REAC	REAC:R-HSA-14...	Extracellular matrix organization	7.823×10^{-12}
8	REAC	REAC:R-HSA-14...	Collagen formation	4.683×10^{-5}
9	REAC	REAC:R-HSA-21...	Integrin cell surface interactions	4.763×10^{-3}
10	WP	WP:WP3888	VEGFA-VEGFR2 signaling	3.569×10^{-4}
11	KEGG	KEGG:04141	Protein processing in endoplasmic reticulum	6.489×10^{-5}
12	KEGG	KEGG:04510	Focal adhesion	1.634×10^{-2}

C

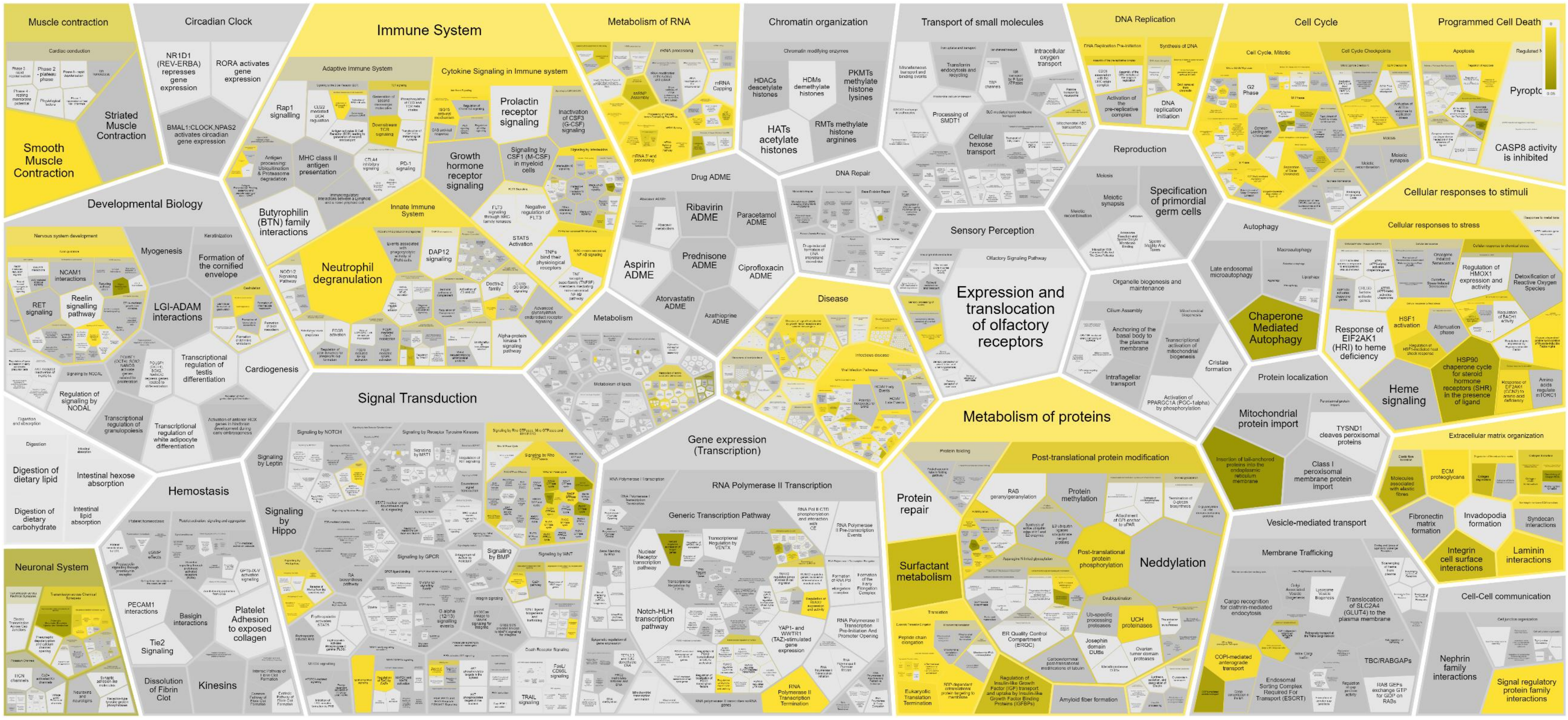

Figure S2

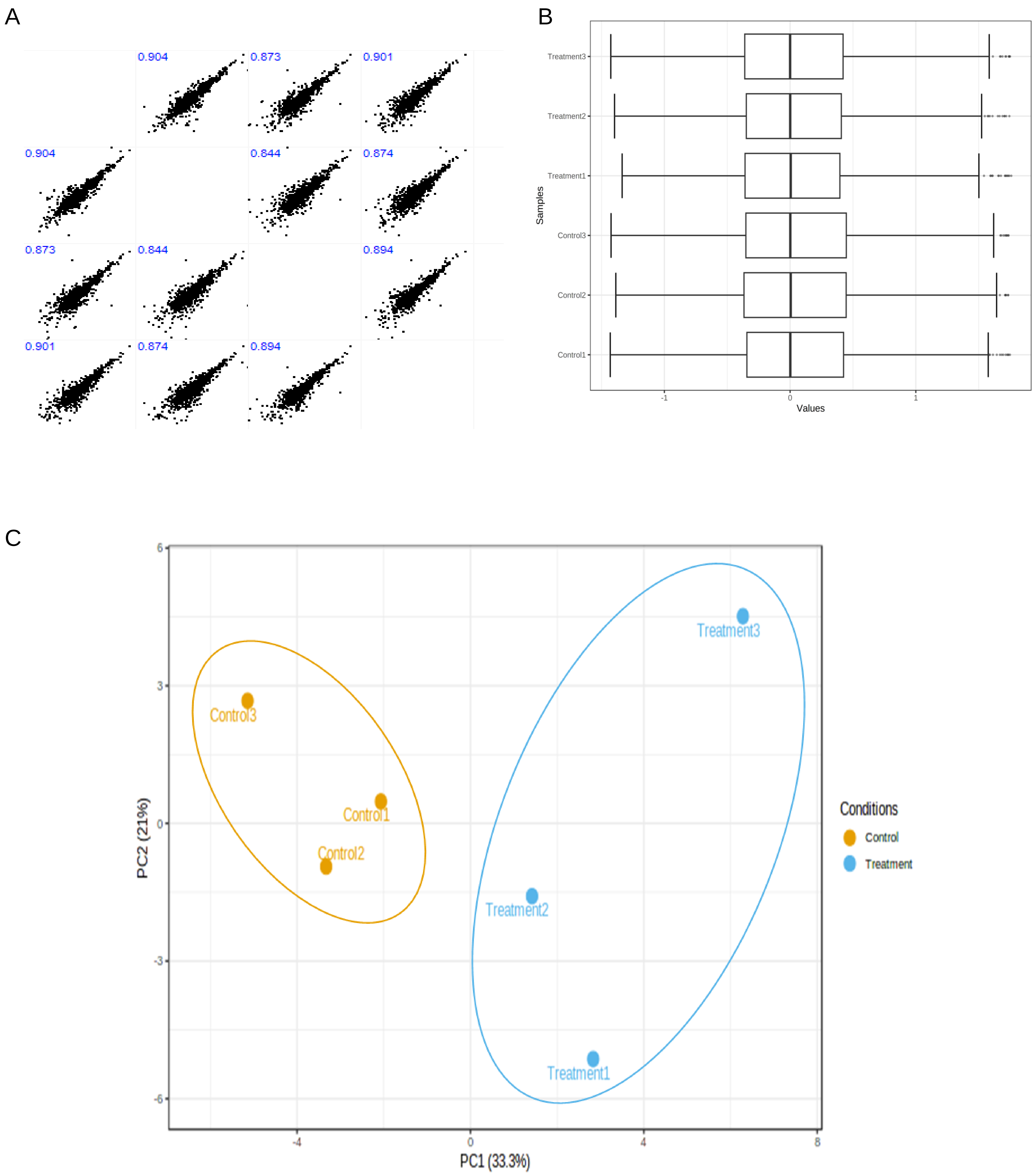
A
0.904
0.873
0.901
0.904
0.844
0.874
0.873
0.844
0.894
0.901
0.874
0.894
B
Treatment3
Treatment2
Treatment1
Control3
Control2
Control1
Samples
-1
0
1
Values
C
6
3
0
-3
-6
PC2 (21%)
-4
0
4
8
PC1 (33.3%)
Control3
Control1
Control2
Treatment3
Treatment2
Treatment1
Conditions
Control
Treatment

Figure S3

Figure S1A represents the classification of the High-Grade Meningioma Sample and control samples of two proteomics datasets PXD007073 and PXD014852; 1B decipher the volcano plot showing the differentially expressed significant proteins with fold change 2 and p-value 0.05 and 1C shows the distribution plot of number of proteins against fold change for all the three datasets

Figure S2A shows the list of 38 common concordant proteins/genes having a p-value less than 0.05 and showing a similar upward or downward trend in all the proteomics and transcriptomics datasets using a stacked bar plot; 2B illustrates the list of top mapped pathways with the DEPs as an input list against Gene Ontology (Molecular Functions), KEGG, Reactome and Wiki Pathway databases and 2C portrays the hierarchical pathways after overrepresentation analysis of the DEPs in Reactome.

Figure S3A represents the correlation plot between the samples; 3B shows the post-normalized sample-wise box plots and 3C shows the PCA plot with clustering between the Control and Cpd22 treated samples in biological replicates.
